# A method for measuring investigative journalism in local newspapers

**DOI:** 10.1073/pnas.2105155118

**Published:** 2021-07-19

**Authors:** Eray Turkel, Anish Saha, Rhett Carson Owen, Gregory J. Martin, Shoshana Vasserman

**Affiliations:** ^a^Graduate School of Business, Stanford University, Stanford, CA 94305

**Keywords:** journalistic impact, local news, machine learning

## Abstract

Major changes to the operation of local newsrooms—ownership restructuring, layoffs, and a reorientation away from print advertising—have become commonplace in the last few decades. However, there have been few systematic attempts to characterize the impact of these changes on the types of reporting that local newsrooms produce. In this paper, we propose a method to measure the investigative content of news articles based on article text and influence on subsequent articles. We use our method to examine over-time and cross-sectional patterns in news production by local newspapers in the United States over the past decade. We find surprising stability in the quantity of investigative articles produced over most of the time period examined, but a notable decline in the last 2 y of the decade, corresponding to a recent wave of newsroom layoffs.

Local newsrooms provide an array of reporting ranging from groundbreaking investigations to local sports coverage and community event announcements. As emerging technologies shift news consumption to different media, local newsrooms are being forced to adjust. Since 2004, there have been nearly 1,800 newspaper closures in the United States (1), along with dozens of ownership changes and steady declines in overall staffing (2). Although these changes have inspired extensive public discussion about the role of news reporting in a democratic society, there has not been a systematic review of the changes in the production of news that took place throughout this period.

In this article, we focus on measuring the investigative content of newspaper coverage. Investigative journalism—reporting that uncovers new information of public interest, and which often requires deep local knowledge and newsroom investment—is one of the most important public functions of the press (3). Journalism scholars have long raised concerns that this kind of content is likely to be undersupplied in a competitive news marketplace (4)—a worry that is exacerbated by the steep declines in advertising revenues that newspapers have faced since the mid-2000s (5). Historically, the emergence of an independent “watchdog” press depended on the growth of newspaper advertising revenues (6, 7). The disappearance of ad revenues in recent decades might therefore be expected to imperil the continued production of investigative content.

Understanding what the changes in the news industry mean for investigative content, however, requires some measure of investigativeness. Measuring investigative content is challenging, because by definition, investigative articles bring to light new information that was not previously public. Clustering methods, counts of entities or predetermined phrases, and latent topic models, which work well for labeling fixed categories of media coverage such as wars, pandemics, sports or weather and have been employed extensively in previous work (8–10), are for this reason, ill-suited to measuring the production of investigative news. To date, approaches to measuring investigative content have largely relied on human coders or keyword searches (e.g., refs. 4 and 11)—approaches that are valuable but do not scale well to the evaluation of large corpora of news stories over a long time span. To address this measurement challenge, we develop a classification algorithm which mixes supervised and unsupervised learning approaches to identify investigative news stories. Our classifier is trained to predict investigativeness based on an article’s impact on topics discussed in future news stories, and text content. The output of our classifier, which is the predicted probability that a given article is investigative (which we call the “score” or p throughout the paper), is used as the evaluation criterion for our analysis.

## Materials and Methods

Our classifier relies on a comprehensive corpus of news articles published by local newspapers across the United States over the past 10 y. Drawing from an archive provided by NewsBank, a news database that collects and archives digital versions of articles from newspapers, we collect the full text and metadata for articles published between 2010 and 2020 by a selection of 50 newspapers that are located in different regions of the United States and have a history of producing investigative content.

In order to train the classifier, we processed the raw text and metadata for each article to generate a rich set of descriptive features that are informative about investigative content. We first built a document frequency matrix of n-grams (words and two-word phrases used in each article), which we used to create high-dimensional representations of each article using a pretrained word embedding model (12). Second, we extracted custom features measuring the occurrence of specific groups of terms that are known to be common in investigative writing (e.g., mentions of the Freedom of Information Act, audits, and court cases) (4). Finally, we trained an unsupervised document influence model of each newspaper’s articles on topics discussed in the subsequent month, and used the measured influences of each article as additional features, which provides the classifier additional information beyond the text alone. Document influence models have been used in previous work to evaluate scholarly impact of scientific articles (13). In our context, they help us identify articles that had a measurable effect on public discourse and future news stories—a prevalent characteristic of investigative news.

Using the full set of features as inputs, we trained a neural network model to predict investigative content. We split our data into three groups for training and testing: Articles published between 2010 and 2017 were used for training (n=5,005,696); articles published in 2018 were used for validation and hyperparameter tuning (n=511,834); and articles published in 2019 (n=409,233) were used for testing only. For our training, we used 562 articles that were labeled as “investigative” because they 1) won first place or runner-up for a relevant journalism award; 2) were entered into the database of the Investigative Reporters and Editors (IRE) for a regular IRE contest; or 3) were hand-selected by a team of reporters for the weekly newsletter *Local Matters*, which showcases investigative reporting from local newspapers’ front pages. The validation and test sets had 213 and 119 labeled investigative articles, respectively. Additional details on our data and model are provided in *SI Appendix*.

## Results

### Validation.

We show that our classifier does well at identifying several hallmarks of investigative quality. First, our classifier successfully predicts articles in unseen data that were handpicked for the *Local Matters* newsletter and/or were ultimately recognized with investigative journalism awards. Using a threshold value of p=0.9 in the test set, our model correctly identifies 80/119 award winners (for a recall value of 0.66), classifies 4,218 other non−award-winning articles as investigative, and classifies 404,894 articles as not investigative.

**Fig. 1.**
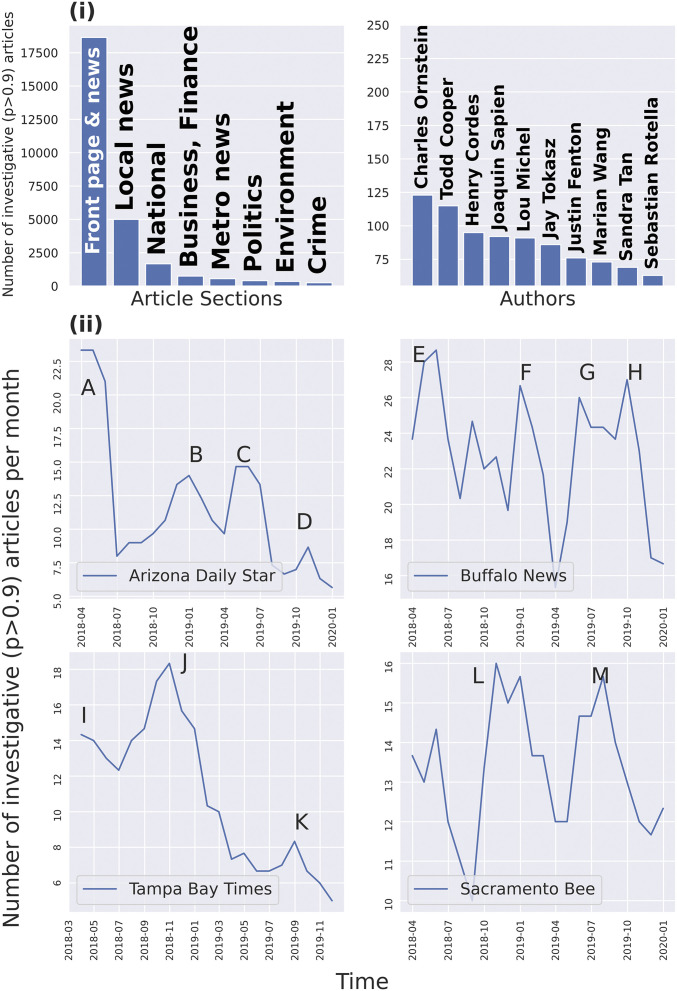
Validation and case studies. (*i*) Most-occurring section names and authors in predicted investigative articles, among articles for which we have section name and author information. (*ii*) Case studies in unseen (post-2018) validation and test data. We identify investigative articles on various topics. A: Rich Rodriguez and Don Shooter scandals; B: Tucson housing crisis; C, D: US/Mexico border wall and family separations along the border; E: Buffalo Water Authority and Percoco corruption cases; F, G, H: Buffalo Diocese and Boy Scout Organization sex scandals; I, K: foster care in Florida, and political campaign spending; J: use of DNA evidence in criminal courts; L: California Camp Fire; M: Gilroy mass shooting, and criminal investigations around the Golden State Killer case. Links to all articles are provided in *SI Appendix*.

Our classifier systematically identifies highly productive authors and assigns high average scores to sections and outlets that specialize in investigative work.* Fig. 1, *i* presents the authors and section names with the highest numbers of articles that are predicted to be investigative by the classifier. Although there is significant variation in naming conventions across newspapers, front page news sections feature the most investigative articles by a large margin, followed by local/state and national sections. Similarly, the leading authors are all distinguished investigative journalists with lengthy portfolios of investigations spanning fraud, corruption, prosecutorial misconduct, environmental hazards, and more.

Furthermore, although our classifier is trained on a narrowly defined set of award-winning investigative articles, it is able to correctly classify articles that are clearly investigative in nature, but that did not receive a “winning” label in our dataset. To demonstrate this, in Fig. 1, *ii*, we plot the count of articles that received a score higher than 0.9 in the validation and test data (post-2018) for four newspapers. For each of the peaks plotted, we examined the articles which contributed to the peak. Although only one of the articles counted in Fig. 1, *i* was tagged as an “award winner,” the peaks overwhelmingly corresponded to investigative stories on topics such as crises in housing, gun rights, family separation, sex abuse, corruption, and crime. Our classifier also reliably identifies articles that are part of a multipart series—a common format for investigative reporting that requires a large fixed investment. Some examples of investigative series plotted in Fig. 1, *ii* include 48 articles published in 2018–2019 investigating sexual abuse in the Buffalo Diocese and seven articles investigating Florida foster care facilities.

### Descriptive Analysis.

As an illustration of the utility of our dataset, we examine broad trends in the production of investigative news as it relates to changes in newspaper industry structure and staffing.

**Fig. 2.**
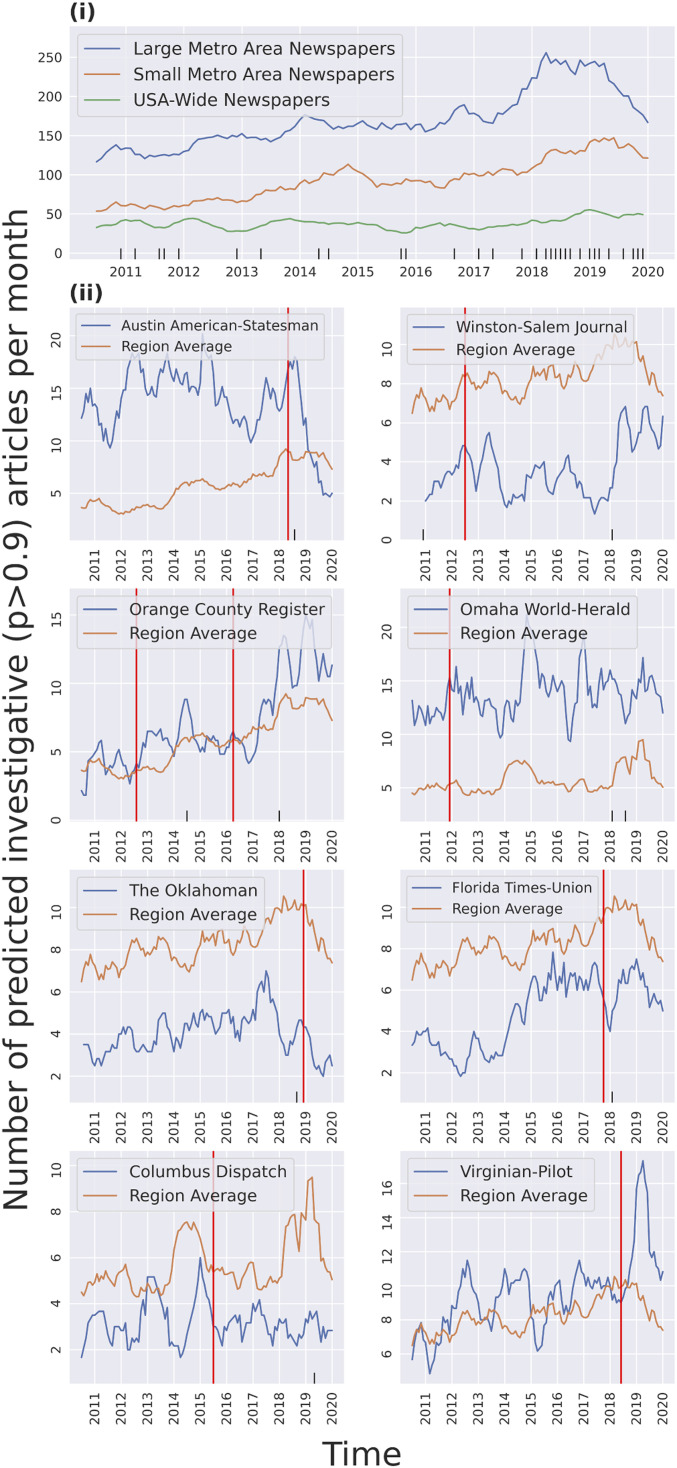
Descriptive analysis. Major layoff events are marked in black on the *x* axis. Plotted values are 6-mo rolling averages. (*i*) Counts of articles that have a high score (*p* > 0.9), grouped by newspaper origin. Twenty-eight newspapers in our dataset originated from large metropolitan areas (>1 million metro population), 20 are from small metropolitan areas (<1 million population), and 2 were published online nationally. (*ii*) Eight newspapers that have been acquired by “investment” firms according to the UNC newspapers database (1). Red lines represent the date of the ownership change.

Fig. 2, *i* shows the overall levels of our measure, aggregated by metro area size. We split the sample of newspapers into three groups: small-metro newspapers, large-metro newspapers, and two specialist national online-only publications that focus on investigative content (ProPublica and the Center for Public Integrity). Perhaps surprisingly, given the turmoil and consolidation in the news industry during this period, we find an overall upward trend in the output of investigative stories for most of the period. Regression analysis on a monthly time trend reveals a coefficient of 0.85 (SD = 0.08) for large-metro, 0.7 (SD = 0.04) for small-metro, and 0.08 (SD = 0.025) for national outlets. The average share of news stories that are predicted to be investigative is 0.7% and 0.5% in large and small metro newspapers, respectively, also with small positive time trends (($6.7\times 10^{-5}$and $4.8\times 10^{-5}$, with SD < $10^{-6}$ in both).

However, Fig. 2, *i* also shows a precipitous drop in output starting in 2019, concentrated at the large-metro papers. The post-2019 monthly time trend coefficients for large-metro, small-metro, and national outlets are −7.7 (SD = 1.32), −4.2 (SD = 1.38), and 0.36 (SD = 0.65), respectively. This drop coincides with a wave of layoff events (plotted at the bottom of Fig. 2, *i*) that began in mid-2018 and continued into 2019.

We next look at acquisitions of newspapers by investment groups: hedge funds and private equity funds. Some scholars have argued that such groups place more weight on financial profitability relative to community benefits (1), leading to worries of shrinking investments in investigative journalism in investment group−owned newsrooms.

Fig. 2, *ii* shows time series plots of our measure at the monthly level for eight papers in our dataset that changed ownership into the “investment group” category.^†^ We find limited evidence that acquisitions of papers by investment companies led to sustained declines in the output of investigative content. Regression analysis using monthly time trends, regional averages, and newspaper ownership status in these eight newspapers reveals that the number of investigative articles per month decreases by 0.22 (SD = 0.485) after an ownership change. Overall, a strong relationship is not visible; while ownership changes are followed by drops in our metric in some cases, most newspapers have no noticeable change in their production of investigative articles. However, we also note that layoff events, which often accompany acquisitions, are predictive of declines in our metric. The outlet-level plots in Fig. 2, *ii* suggest that not all layoffs are created equal; a buyout offered to all 200+ employees of the Austin American-Statesman in 2018 was followed by a precipitous decline in our measure of investigative news at that paper, whereas more-limited layoffs at the Florida Times-Union, concentrated among part-time employees, did not noticeably shift the paper’s average output in our measure relative to its peers.

## Discussion

This descriptive evidence offers some hope that the consequences of changes in the news industry, on one of its most important outputs, may not be as bad as feared. However, it also suggests the caution that downsizing and restructuring are slow-moving processes, and we may not have seen their full impact yet. Our complete article-level dataset of 5.9 million articles with metadata and our predicted investigativeness scores is publicly available. We expect that this dataset will be useful to researchers interested in a variety of questions on the organization of the news industry and its public consequences.

## Supplementary Material

Supplementary File

## Data Availability

The article-level dataset with authors, titles, newspaper names, dates, neural network inputs, predicted scores, and award winning status has been deposited in Harvard Dataverse (10.7910/DVN/HSZ2QL).

## Footnotes

*Information about author identities is not an input to the predictive model, and thus this constitutes a validation check on the model predictions.

^†^We relied on the University of North Carolina (UNC) Center for Innovation and Sustainability in Local Media’s database (1) of ownership to identify newspapers that changed to the “investment group” category in this period, and then identified acquisition dates from contemporaneous news reports.
