# Supplementary material for: A method for measuring investigative journalism in local newspapers
Source: Proc Natl Acad Sci U S A. 2021 Jul 19;118(30):e2105155118. doi: 10.1073/pnas.2105155118 (PMC8325361; doi:10.1073/pnas.2105155118)
Supplement: Supplementary File [file pnas.2105155118.sapp.pdf]

PNAS
www.pnas.org

Supplementary Information for

A Method for Measuring Investigative Journalism in Local Newspapers

Eray Turkel, Anish Saha, Rhett Carson Owen, Gregory J. Martin, Shoshana Vasserman

Eray Turkel
E-mail: eturkel@stanford.edu

This PDF file includes:

Supplementary text
SI References

Supporting Information Text

Extended Materials and Methods

The full data set used for this study, in addition to code that can be used to build the dynamic influence model and the word embedding representations is publicly available (1).

Data Collection. The data set utilized for the analysis described in this paper was provided by NewsBank, a news database resource that provides a collection of millions of articles and media publications from thousands of different sources. We collected all available articles from 50 US newspapers among those that have historically won journalistic awards, choosing a geographically diverse set of newspapers. The articles from these sources were systematically queried, and the resultant data was reformatted and aggregated into the finalized raw data set. After removing advertisements and obituaries, the final data set consisted of 5,926,763 articles, 894 of which were winners or nominees of journalistic awards. We use all the articles between Jan. 1, 2010- Dec. 31, 2017 as our training set, Jan. 1- Dec. 31, 2018 as our validation set, and Jan. 1 - Dec. 31 2019 as our test set. The number of articles in the training, validation, and test sets were $5005696, 511834$ and 409233, with $562, 213$ and 119 award winning articles, respectively in each group.

Data Preprocessing. The first preprocessing step was reducing the words in the article text to their linquistic stems, and tokenizing the text into n-grams $[n \in \{1, 2\}]$ to generate a document-frequency matrix based on the 20,000 most commonly found n-grams within each newspaper.

Next, for every newspaper, we split our corpus into monthly groups and trained a dynamic document influence model with 8 topics. This method has previously been used to evaluate the scholarly impact of scientific articles (2, 3). Influence scores, a measure of how an article affected next months articles on every topic, were obtained from this model. Each dynamic LDA topic for the 50 newspapers in our data set consisted of [20,000 x 120] matrices, where every row was a probability distribution over the terms in the vocabulary for every month. For every newspaper, these topic distributions were averaged over time, creating 50 distinct average distribution vectors for 8 topics. A greedy optimization algorithm was then used to match these topics across newspapers (4) based on their shared vocabulary, creating 8 topic clusters. The influences of articles on each topic were labeled according to these clusters to achieve consistency across different newspapers.

A separate step was to perform feature extraction on the text data in order to retrieve further information that could provide helpful information for our classification model. We conducted a human-designed feature extraction process, which tagged certain common article behaviors as potentially influential: any indication or mention of involvement in a series of articles, words related to investigative acts and court cases, inclusion of statistical, anecdotal, legislative data, references to contemporary investigations, and the number of words and sentences in the article.

Further steps were taken to extract useful information from the article sections. Namely, to capture the fact that some sections in newspapers were more likely to produce investigative articles compared to others (e.g. metro news versus sports), for every newspaper, we generated a document-frequency matrix using the words used in the section names of every article. We used this document-frequency matrix to train two separate gradient-boosting classification models on the training set. The first one was trained to predict whether a given section had any award winning articles at all, using section names (i.e., the ground truth label was positive for every article belonging to a section that had an award winning article). The second one was trained to predict award winning articles using section names (i.e., the ground truth label was positive only for award winning articles). The outputs (probabilities) from these two models were used as additional features for our final classification task.

Further, we used the pre-trained word embedding model FastText (5–7) which consisted of 1 million word vectors trained on Wikipedia, UMBC webbase corpus, and statmt.org news data set to create 300-dimensional latent representations for articles in our data set. For every article, we used the tf-idf weighted average of the word embeddings of the constituent words to generate article embeddings (8, 9).

Our final set of 320 features for each news article included the 300-dimensional article embeddings, 10 features from the document influence model (8 features representing the article's influence score on 8 topics, the sum of the influence values on all topics, and the maximum of the 8 influence scores), 2 features which are the probabilities extracted from the section-name based classifier described above, and 8 features from our human-designed feature extraction process (number of words, number of sentences, mention of a series of articles, number of references to other parts of an article series, accountability-related word count, investigation-related word count, mentioned investigation duration, mention of any investigation documents).

Classifier Model. We used a three-layer neural network with the focal loss function (10), that has been found to be useful in classification tasks involving imbalanced data sets. Hyperparameter experiments were run to find the best model architecture using a grid search over the number of nodes within every layer, the regularization parameters, and the parameters of the focal loss function. When choosing the final model to deploy, we used a holistic approach and analyzed performance on the validation data set. We prioritized recall over precision, and investigated the false positives belonging to non-investigative sections within every newspaper. Our final neural network architecture is a three layer model with [256,128,128] nodes with RelU activation, and the model was trained with dropout layers between every hidden layer with respective probabilities [0.4,0.4,0.6]. Our focal loss function uses the parameter values $\alpha = 0.9999, \gamma = 3.0$.

On the test data set, using a threshold value of 0.5, our model correctly identifies $92/119$ award winning investigative articles. Our model also predicts that 8949 articles who did not win awards are investigative, and classifies the remaining 400163 articles as not investigative. With a threshold value of 0.9, the model correctly identifies $80/119$ award winners, predicts

4218 other non-award winning articles are investigative, and classifies 404894 articles as not investigative. The test AUC-ROC achieved by the model is 0.99, and AUC-PR is 0.02.

List of Newspapers. Full list of newspapers in our data set is provided below. For each newspaper, we queried all articles on Newsbank between Jan 1, 2010 - Dec 31, 2019. For some newspapers, articles were only available for a subset of this period, which we specify below.

Albuquerque Journal (NM)
Anchorage Daily News (AK, Jan 2010-Jul 2014, Jan 2015-Jun 2015, May 2016-Dec 2019)
Arizona Daily Star (AZ)
Austin American-Statesman (TX)
Baltimore Sun (MD)
Buffalo News (NY)
Center for Public Integrity (USA)
Charlotte Observer (NC)
Chicago Sun-Times (IL)
Columbus Dispatch (OH)
Commercial Appeal (TN, all except Dec 2016-Feb 2017)
Dallas Morning News (TX, Jan 2010-Nov 2013)
Denver Post (CO)
Detroit News (MI)
East Valley Tribune (AZ, all except Feb 2017)
El Paso Times (TX)
Florida Times-Union (FL)
Fresno Bee (CA)
Houston Chronicle (TX)
Idaho Statesman (ID)
Kansas City Star (MO, Jan 2010-Sep 2018)
Los Angeles Times (CA, Sep-Dec 2013, Dec 2014-Jan 2016, Jul-Dec 2016, Feb 2017-Mar 2018, Oct 2018-Dec 2019)
Las Vegas Sun (NV)
Lexington Herald-Leader (KY)
Miami Herald (FL)
Milwaukee Journal Sentinel (WI)
Oklahoman (OK)
Omaha World-Herald (NE)
Orange County Register (CA)
Philadelphia Inquirer (PA)
Pittsburgh Post-Gazette (PA)
Plain Dealer (OH)
ProPublica (USA)
Sacramento Bee (CA)
San Antonio Express-News (TX)
San Diego Union-Tribune (CA, Jan 2010-Feb 2012, May 2015-Dec 2019)
San Jose Mercury News (CA, Jan 2010-Jun 2016)
Seattle Times (WA)
San Francisco Chronicle (CA)
Spokesman-Review (WA)
St. Louis Post-Dispatch (MO)
Star Tribune: Newspaper of the Twin Cities (MN)
Tampa Bay Times (FL, Dec 2011-Dec 2019)
The Gazette (CO)
The Oregonian (OR)
The Blade (OH, May 2013-Dec 2019)
The Virginian-Pilot (VA)
The Wichita Eagle (KS, Dec 2010-Dec 2019)
Winston-Salem Journal (NC)
Wisconsin State Journal (WI)

List of all custom words and phrases extracted. The set of features extracted are derived from the extensive description of keywords used and characteristics of investigative news stories in (11). Specifically: (i) investigative stories are very frequently part of a series, and (ii) they often uncover new information on a topic of public interest that potentially someone in power would like to conceal, hence the keywords below are frequently used in investigative stories as described in (11).

Audit, FOIA, freedom of information act, court case, abuse, corruption,crime, exploitation, injustice, misconduct, misdeed, misuse, offense, wrongdoing, debasement, delinquency, desecration, fault, misapplication, mishandle, mishandling, mismanage, perversion, bribe, extortion, fraud, malfeasance, nepotism, crookedness, demoralization, jobbery, misrepresent, payoff, payola, shady, skimming, unscrupulous, venality, profiteer, blackmail, deceit, hoax, scam, artifice, barratry, cheat, chicane, con, dupe, duplicity, guile, imposture, sham, treachery, swindle, derelict, immorality, impropriety, malpractice, misbehavior, mischief, mischevious, misdemeanor, transgression, botch, neglect, abandon, disuse, mistreat, imperil, jeopardize, endanger, abduct, divert, embezzle, blackmail, accuse, allege, arraign, arrest, attack, blame, charge, complain, denounce, implicate, indict, prosecute, sue, summon, apprehend, betray, censure, frame, impeach, impute, incriminate, risk, steal, records OR documents OR analysis + showed OR found OR revealed OR uncovered OR documented OR discovered, X-(month OR year) long investigation, X'th part of series.

List of Journalistic Awards Used. The Sidney Hillman Foundation Awards, NLA Awards (Previously ASNE), ASNE Awards, National Press Foundation Awards, Dart Awards for Excellence in Coverage of Trauma, Sigma Delta Chi Awards, IRE Awards, Local Matters Picks (Sep 2017 onwards), Pulitzer Awards, OJA Awards, Goldsmith Awards, Mike Berger Award, Paul Tobenkin Memorial Award, George Polk Awards, Overseas Press Club Awards, Society of Professional Journalists Sigma Delta Chi Awards.

Additional Details on the Main Text

List of Articles Identified in Figure 2: Post-2018 Case Studies. A: Investigations on Rich Rodriguez and Don Shooter scandals (12, 13) B: Series on Tucson housing crisis (14) C, D: Series on the Mexican border wall and family separations along the border (15, 16) E: Series on Buffalo Water Authority and Percoco Corruption Cases (17, 18) F, G, H: Investigations on the Buffalo Diocese and Boy Scout Organization sex scandals (19–21) I: Series on foster care in Florida, and political campaign spending (22, 23) J: Series on the use of DNA evidence in criminal courts (24) K: Series on Florida foster care facilities (25) L: California Camp Fire (26) M: Gilroy mass shooting, and criminal investigations around the Golden State Killer case (27, 28).

References

1. E Turkel, A Saha, CR Owen, JG Martin, S Vasserman, Data Set: Measuring Impactful Reporting in Local Newspapers (https://doi.org/10.7910/DVN/HSZ2QL) (2021) [Online; accessed 24-March-2021].
2. A Gerow, Y Hu, J Boyd-Graber, DM Blei, JA Evans, Measuring discursive influence across scholarship. *Proc. national academy sciences* **115**, 3308–3313 (2018).
3. S Gerrish, DM Blei, A language-based approach to measuring scholarly impact in *ICML*. (2010).
4. D Newman, A Asuncion, P Smyth, M Welling, Distributed algorithms for topic models. *J. Mach. Learn. Res.* **10** (2009).
5. P Bojanowski, E Grave, A Joulin, T Mikolov, Enriching word vectors with subword information. *arXiv preprint arXiv:1607.04606* (2016).
6. A Joulin, E Grave, P Bojanowski, T Mikolov, Bag of tricks for efficient text classification. *arXiv preprint arXiv:1607.01759* (2016).
7. A Joulin, et al., Fasttext.zip: Compressing text classification models. *arXiv preprint arXiv:1612.03651* (2016).
8. Q Le, T Mikolov, Distributed representations of sentences and documents in *International conference on machine learning*. (PMLR), pp. 1188–1196 (2014).
9. C De Boom, S Van Canneyt, T Demeester, B Dhoedt, Representation learning for very short texts using weighted word embedding aggregation. *Pattern Recognit. Lett.* **80**, 150–156 (2016).
10. TY Lin, P Goyal, R Girshick, K He, P Dollár, Focal loss for dense object detection in *Proceedings of the IEEE international conference on computer vision*. pp. 2980–2988 (2017).
11. JT Hamilton, *Democracy's Detectives: The Economics of Investigative Journalism*. (Harvard University Press, Cambridge, Mass.), (2016).
12. Arizona house expels yuma rep. don shooter after sexual-harassment findings (https://tucson.com/news/local/arizona-house-expels-yuma-rep-don-shooter-after-sexual-harassment-findings/article_3f22e961-be34-5410-b27e-a878e6396844.html) (2018) [Online; accessed 15-March-2021].
13. C Schmidt, Breakdown of sexual harassment claims against ex-wildcats coach rich rodriguez (https://tucson.com/news/local/breakdown-of-sexual-harassment-claims-against-ex-wildcats-coach-rich-rodriguez/article_94e9ed68-f0b0-11e7-a8ec-c3ea7f93a46e.html) (2018) [Online; accessed 15-March-2021].
14. E Bregel, Low-income housing crisis takes toll on tucson renters (https://tucson.com/low-income-housing-crisis-takes-toll-on-tucson-renters/article_ab1605a8-e39f-11e8-9618-5363f9cf82f5.html) (2018) [Online; accessed 15-March-2021].

15. C Prendergast, Families separated along arizona border allege suffering in claims against us (https://tucson.com/news/local/families-separated-along-arizona-border-allege-suffering-in-claims-against-us/article_4e14b47d-e48c-56ce-a6ec-30b8e18a20e4.html) (2019) [Online; accessed 15-March-2021].
16. C Prendergast, Star investigation: Arizona's border wall is being built with anti-drug money. but it won't stop hard drugs (https://tucson.com/news/local/star-investigation-arizonas-border-wall-is-being-built-with-anti-drug-money-but-it-wont/article_675670d4-f10c-11e9-ab2a-fb568a3a9bdf.html) (2019) [Online; accessed 15-March-2021].
17. M Spina, New golden parachute protects erie county water authority boss (https://buffalonews.com/news/local/new-golden-parachute-protects-erie-county-water-authority-boss/article_395fe34c-ccdb-529d-af1d-b750614cf66c.html) (2018) [Online; accessed 15-March-2021].
18. T Precious, Percoco conviction carries political fallout for cuomo; ex-top aide found guilty of corruption (https://buffalonews.com/news/local/percoco-corruption-conviction-is-a-political-blow-for-cuomo/article_fed742d6-6e1d-5711-8776-73779119ce5d.html) (2018) [Online; accessed 15-March-2021].
19. J Tokasz, Bishop malone: There's nothing being hidden (https://buffalonews.com/news/local/bishop-malone-theres-nothing-being-hidden/article_9b84e8ea-d55c-55d9-8cc2-65326ee74064.html) (2018) [Online; accessed 15-March-2021].
20. M McAndrew, Buffalo area boy scout leaders linked to sex abuse involving children (https://buffalonews.com/news/local/crime-and-courts/buffalo-area-boy-scout-leaders-linked-to-sex-abuse-involving-children/article_76d0ca47-42fa-5cf1-b5e9-46d3801e798c.html) (2019) [Online; accessed 15-March-2021].
21. D Herbeck, Who is father ryszard biernat, and why would he secretly record bishop malone? (https://buffalonews.com/news/local/who-is-father-ryszard-biernat-and-why-would-he-secretly-record-bishop-malone/article_3e50ad79-8b94-5fda-8d6e-3c103ae8f7b8.html) (2019) [Online; accessed 15-March-2021].
22. C O'Donnell, E Murray, C Humburg, , et al., Zombie campaigns (https://projects.tampabay.com/projects/2018/investigations/zombie-campaigns/spending-millions-after-office/) (2018) [Online; accessed 15-March-2021].
23. C O'Donnell, Foster care failures uncovered in death of 2-year-old jordan belliveau (https://www.tampabay.com/news/publicsafety/foster-care-failures-identified-in-state-report-on-the-death-of-2-year-old-largo-boy-20190116/) (2018).
24. LL Anton, Blood and truth (https://projects.tampabay.com/projects/2018/narratives/blood-and-truth/) (2018) [Online; accessed 15-March-2021].
25. C O'Donnell, Problem foster kids could be locked up in 'secure' facility under new plan pushed by tampa bay child welfare agency (https://www.tampabay.com/news/hillsborough/2019/09/09/problem-foster-kids-could-be-locked-up-in-secure-facility-under-new-plan-pushed-by-tampa-bay-child-welfare-agency/) (2019) [Online; accessed 15-March-2021].
26. R Sabalow, , et al., This fire was outrunning us: Surviving the camp fire took bravery, stamina and luck (https://www.sacbee.com/news/california/fires/article221980270.html) (2018) [Online; accessed 15-March-2021].
27. J Ding, S Stanton, Gunman in gilroy mass shooting bought 'assault-type rifle' legally in nevada, police say (https://www.sacbee.com/news/california/article233261797.html) (2019) [Online; accessed 15-March-2021].
28. T Bizjak, B Egel, Sacramento county da making a name for groundbreaking dna investigations (https://www.sacbee.com/news/local/crime/article232163062.html) (2019) [Online; accessed 15-March-2021].
